# Variable phenotypes and outcomes associated with the *MMACHC* c.609G>A homologous mutation: long term follow-up in a large cohort of cases

**DOI:** 10.1186/s13023-020-01485-7

**Published:** 2020-08-03

**Authors:** Ruxuan He, Ruo Mo, Ming Shen, Lulu Kang, Jinqing Song, Yi Liu, Zhehui Chen, Hongwu Zhang, Hongxin Yao, Yupeng Liu, Yao Zhang, Hui Dong, Ying Jin, Mengqiu Li, Jiong Qin, Hong Zheng, Yongxing Chen, Dongxiao Li, Haiyan Wei, Xiyuan Li, Huifeng Zhang, Min Huang, Chunyan Zhang, Yuwu Jiang, Desheng Liang, Yaping Tian, Yanling Yang

**Affiliations:** 1 Department of Pediatrics, Peking University First Hospital, Beijing, 100034 China; 2 Research Center for Translational Medicine, Medical Innovation Research Division of Chinese PLA General Hospital, Beijing, 100853 China; 3 Department of Pediatric Surgery, Peking University First Hospital, Beijing, China; 4 Department of Pediatrics, People’s Hospital of Peking University, Beijing, China; 5 Department of Pediatrics, First Affiliated Hospital of Henan University of Traditional Chinese Medicine, Zhengzhou, China; 6 Department of Endocrinology and Inherited Metabolic, Henan Children’s Hospital, Zhengzhou, China; 7 Precision Medicine Center, General Hospital of Tianjin Medical University, Tianjin, China; 8 Department of Pediatrics, Hebei Medical University Second Hospital, Shijiazhuang, China; 9 Similan Clinic, Beijing, China; 10 School of Life Sciences, Central South University, Changsha, 410013 China

**Keywords:** Cobalamin C deficiency (*cblC*), The *MMACHC* gene, C.609G > A, Methylmalonic acidemia and homocysteinemia

## Abstract

**Background:**

Cobalamin C deficiency (*cblC*) caused by the *MMACHC* mutations is the most common type of the disorders of intracellular cobalamin metabolism. While the c.609G > A mutation is most frequent in Chinese *cblC* patients, its correlation with phenotype has not been delineated. Here we aim to investigate the factors affecting variable phenotypes and outcomes associated with the *MMACHC* c.609G > A homologous mutation in 149 Chinese cases to have implications for treatment and prevention.

**Methods:**

We assessed 149 *cblC* patients caused by *MMACHC* c.609G > A homozygous mutation. The clinical manifestations, complications, treatment, and outcomes were evaluated; 120 patients were followed-up till December 2019.

**Results:**

Two patients (1.3%) were prenatally diagnosed, treated after birth and consequently showed normal development. In 15 patients (10.1%) detected by newborn screening, 10 were treated at the age of 2 weeks and showed normal development, while the other 5 were treated after onset and showed neurologic disorders. All 132 clinically diagnosed patients (88.6%) developed symptoms at age from few minutes after birth to 72 months. Among them, 101 (76.5%) had early-onset (before the age of 12 months) and 31 (23.5%) had late-onset (after the age of 12 months). Totally 5 patients died and 24 were lost to follow-up. Of the 132 clinical diagnosed patients, 92 (69.7%) presented with developmental delay, 65 (49.2%) had seizures, 37 (28.0%) had anemia, 24 (18.2%) had feeding difficulty, 23 (17.4%) had ocular problems, and 22 (16.7%) had hydrocephalus. Compared with the non-developmental delay group, the onset age, the age at treatment initiation and the time from onset to treatment initiation were later in the developmental delay group. Seizure group showed significantly higher urinary methylmalonic acid concentration. During long-term follow-up, plasma total homocysteine (tHcy) levels were significantly higher in patients in the uncontrolled group than those in the seizure-free group.

**Conclusions:**

Most *cblC* patients caused by *MMACHC* c.609G > A homozygous mutation showed early-onset. The clinically diagnosed patients usually showed the presence of irreversible brain disorders. Patients treated from the pre-symptomatic stage showed favorable outcomes. Therefore, newborn screening, prenatal diagnosis and early treatment are crucial and the c.609G > A mutant allele should be listed in the pre-pregnancy carrier screening panel in China.

## Background

The cblC type (*cblC*) of combined methylmalonic acidemia and homocystinuria is the most common defect in the intracellular cobalamin metabolism pathway, characterized by variable and non-specific symptoms especially in childhood [1–3]. An increasing hospitalization with various presentations and a heavy financial burden per hospitalization were observed in Mainland China, while the medical resources were still relatively centralized in some districts, such as Beijing, Shanghai and Guangzhou [4]. All the disorders of intracellular cobalamin metabolism are inherited in an autosomal recessive manner except for cblX, which is inherited in an X-linked manner [5]. In the patients with *cblC*, age of onset ranges from prenatal to adult and clinical manifestations vary from mild to life-threatening [6]. The most common *MMACHC* pathogenic variant previously reported, c.609G > A (p.Trp203X), results in a premature termination codon that is predicted to cause a truncated or absent *MMACHC* protein due to nonsense mediated decay and accounts for 48.1% of mutant alleles in 79 Chinese patients with *cblC* [3]. This variant is listed in the Genome Aggregation Database (gnomAD) with an overall population frequency of 0.004% and has been reported in numerous affected individuals in the homozygous and compound heterozygous state, and there is evidence that the variant may be a founder mutation in the Chinese population [3, 7] .

The *MMACHC* c.609G > A is the hot spot mutation in Chinese patients with early-onset *cblC* [2]. Neurological disorders are common complications of *cblC*, which may result in varying degrees of sequelae [2, 3]. Epilepsy is a frequent symptom in children with early-onset *cblC* [8]. With an improvement in clinical diagnosis, there has been an increase in the number of cases being diagnosed with *cblC*-related epilepsy or other neurological diseases. Patients with *cblC* caused by the *MMACHC* c.609G > A homozygous mutation have been found to show significant differences in phenotypes and outcomes.

In the present study, we carried out a retrospective analysis of 149 Chinese *cblC* patients with the *MMACHC* c.609G > A homozygous mutation who were followed up in Department of Pediatrics, Peking University First Hospital, Beijing. Clinical features, metabolites, diagnosis, process of management and outcomes were reviewed and factors affecting variable phenotypes and outcomes were investigated.

## Methods

### Patients and data collection (Table 1)

From January 1998 to December 2019, 149 patients with *cblC* attributable to *MMACHC* c.609G > A homozygous mutation were diagnosed by gene sequencing at the Peking University First Hospital in China. These patients came from 11 provinces or cities of Mainland China. Five cases died and 24 cases were lost to follow-up. The remaining 120 cases received personalized treatment and regular follow-up. Patients with definite pathogenic variants in genes besides *MMACHC* were excluded.

**Table 1:** General information pertaining to 149 patients caused by the *MMACHC* c.609G > A homozygous mutation

Patients	n	Male		Female	
		n	%	n	%
Diagnosed prenatally and treated at birth	2	1	0.7	1	0.7
Detected by newborn screening	15	7	4.7	8	5.4
Treated at birth	10	6	4.0	4	2.7
Treated after onset	5	1	0.7	4	2.7
Clinically diagnosed	132	73	49.0	59	39.6
Death	5	2	1.3	3	2.0
Regular follow-up	103	60	40.3	43	28.9
Lost to follow-up	24	11	7.4	13	8.7
Total	149	81	54.4	68	45.6

Note: *n* number

General data were collected, including the age at onset and diagnosis, clinical manifestations, family history, treatment and outcomes. Electroencephalography and brain imaging by MRI or CT were performed. Elevated blood propionylcarnitine and urinary methylmalonic acid levels confirmed the diagnosis of MMA. All patients had significantly elevated plasma total homocysteine (tHcy) (34.3–278 μmol/L, normal control ≦15 μmol/L). Moreover, routine blood and urine examination were performed to evaluate liver, renal, and heart function.

### Biochemical examination

Dried blood spots were collected. Blood amino acids, free carnitine, and acylcarnitines were analyzed using liquid chromatography–tandem mass spectrometry (Waters MS/MS system A, 1445–002; API3200, Applied Biosystems, CA, USA), as previously described [9, 10]. Metabolite concentrations were automatically calculated using the Chemoview software [2, 9]. The reference range for propionylcarnitine was 1.00–5.00 μmol/L.

Gas chromatography–mass spectrometry (GC–MS), which was performed on a Shimadzu GCMS-QP2010 system (Kyoto, Japan), was used to analyze urinary organic acids, as previously reported. Mass spectra were obtained by standard electron impact ionization scanning from 50 *m/z* to 500 *m/z*. Data were collected using a GC–MS solution software [11, 12].

Plasma total homocysteine (tHcy) were assessed using fluorescence polarization immunoassay [2].

### Treatment

For patients during acute decompensation, the initial therapy involved intramuscular or intravenous injections of cobalamin (hydroxocobalamin was the first choice at a dose of 1 mg per day), L-carnitine (50–200 mg/kg per day), intravenous fluid therapy with glucose and electrolytes, oral betaine (100–500 mg/kg per day), a high-calorie diet with symptomatic treatment. If hydroxocobalamin was not available, methylcobalamin was used for injection. All patients followed a normal diet. For the patients with methionine deficiency, methionine was supplemented by oral. The long-term treatment was then adjusted depending on the condition of individual patients [2, 13].

### Genomic analyses

Genomic DNA was extracted from peripheral blood of patients and their parents using a TIANamp® Blood DNA Kit (Tiangen Biotech Co. Ltd., Beijing, China), as per manufacturer instructions. Next-generation sequencing (NGS) was performed to screen for mutations (Running Gene Inc., Beijing, China; Berry Genomics Corporation, Beijing, China; Translational Medicine Laboratory, Chinese People’s Liberation Army General Hospital, Beijing, China). The *MMACHC* c.609G > A homozygous mutation was found in 149 patients. Patients with other gene mutations associated with MMA and homocystinemia were excluded.

### Statistical analysis

SPSS 24.0 (SPSS Inc., Chicago, IL, USA) was used for statistical analyses. Analyses using the Shapiro–Wilk method showed that the data did not conform to normal distribution, so the values are expressed as median (quartile), and nonparametric unpaired Mann-Whitney U test was also performed. All tests were set as two-tailed, and *P* < 0.05 indicated statistical significance.

## Results

### Patients (Table 1)

In total, 149 patients with *cblC* attributable to the *MMACHC* c.609G > A homozygous mutation were analyzed. Among them, 81 (54.4%) were males and 68 (45.6%) were females. Two unrelated cases (one male and one female) were prenatally diagnosed based on the family history that their elder siblings had died of *cblC* [14, 15]. *CblC* was diagnosed by the increasing of methylmalonic acid and total homocysteine in amniotic fluid and *MMACHC* c.609G > A homozygous mutation in amniocytes at the gestational age of 20 weeks. No abnormal echo was found in the two fetuses by ultrasound examination. Mild carnitine deficiency, slight methylmalonic aciduria and normal plasma total homocysteine were observed in their mothers. The mothers chose to continue their pregnancy. Oral methylcobalamin (1 mg per day), L-carnitine (1 g per day) and multivitamins were supplemented during their pregnancy to support the growth and development of fetuses. Their blood free carnitine levels were maintained at the normal range. Two patients were treated from the first day of birth. At present, they are aged 3 and 11 years respectively, with normal physical and neurocognitive development. No abnormal finding was found by eye test.

Fifteen cases (10.1%) were detected by newborn screening. Among them, treatment was administered to ten cases (six males and four females; intramuscular cobalamin injection, oral administration of L-carnitine, and betaine) at the age of 15 days. They are currently 2 to 8 years old, with normal psychomotor and physical development. Unfortunately, parents of the other five cases (one male and four females) refused the confirmative testing and presymptomatic treatment. These cases developed symptoms at the age of 1–4 months, including developmental delay in all five cases, hydrocephalus in two cases, and seizures in one case.

### Phenotypic features

One hundred and thirty-two cases (88.6%) were clinically diagnosed. The onset age ranged from a few minutes after birth to age 72 months (median age 3 months). The average time from onset to confirmative diagnosis was 10 months. Early onset (before the age of 12 months) was observed in 101 cases (76.5%), of whom 36 (27.3%) exhibited symptoms during the neonatal period. Late onset (after the age of 12 months) was observed in 31 cases (23.5%). The most common clinical manifestations were developmental delay (69.7%), followed by seizures (49.2%), anemia (28.0%), feeding difficulty (18.2%), ocular problems (17.4%), and hydrocephalus (16.7%) (Table 2). Of the 132 clinically diagnosed patients, strabismus, nystagmus, visual impairment, and maculopathy were observed in 23 patients (17.4%) at their first visit by eye tests. Frequencies were compared between early-onset (*n* = 101) and late-onset (*n* = 31) groups. As shown in Table 2, the frequency of developmental delay was significantly higher in the late-onset group, and the frequency of hydrocephalus was significantly higher in the early-onset group.

**Table 2:** Clinical manifestations of 132 clinical diagnosed *cblC* patients caused by the *MMACHC* c.609G > A homozygous mutation

Clinical manifestations	Cases		Early-onset (***n*** = 101)		Late-onset (***n*** = 31)		***P*** value
	n	%	n	%	n	%	
Developmental delay	92	69.7	63	62.4	29	93.5	**0.003**
Epilepsy	65	49.2	54	53.5	11	35.5	0.053
Anemia	37	28.0	32	31.7	5	16.1	0.073
Feeding difficulty	24	18.2	21	20.8	3	9.7	0.138
Hydrocephalus	22	16.7	21	20.8	1	3.2	**0.018**
Ocular problems	23	17.4	19	18.8	4	12.9	0.399
Vomiting	19	14.4	15	14.9	4	12.9	0.951
Hypotonia/abnormal muscle tone	15	11.4	10	9.9	4	12.9	0.944
Skin lesions	12	9.1	11	10.9	1	3.2	0.319
Lactic acidosis	6	4.5	6	5.9	0	0.0	0.335
Liver damage	5	3.8	5	5.0	0	0.0	0.335
Renal damage	2	1.5	1	1.0	1	3.2	0.427
Cardiomyopathy	0	0.0	0	0.0	0	0.0	
Pulmonary hypertension	0	0.0	0	0.0	0	0.0	

Notes: *n* number; *P* value was calculated by using a chi-square test. *P* < 0.05 was considered as statistically significant

### Follow-up and outcomes

The two cases diagnosed prenatally and 15 cases detected by newborn screening were followed up regularly.

Of the 132 clinical diagnosed cases (Tables 1, 2), 37 were misdiagnosed as nutritional anemia (*n* = 9), cerebral palsy (*n* = 9), hypoxic ischemic encephalopathy (*n* = 8), epilepsy (*n* = 5), pneumonia (*n* = 4), and autism (*n* = 2). The age at diagnosis ranged from 3 days to 101 months (median 6 months). Time delays from the onset to treatment initiation was 7 days to 96 months (median 2 months, average = 12 months). Early onset was observed in 101 cases (76.5%), with symptoms appearing before the age of 1 year. Late onset was observed in 31 cases (23.5%), with symptoms appearing from the age of 14 months to 72 months (median 24 months), and median age of diagnosis is 39 months. Five (3.8%) patients died. Two of them died of intractable epilepsy at the age of 9 months and 10 years, respectively. The three others died of multiple organ failure induced by infection at the age of 3 months, 2 years and 4 years, respectively. Twenty-four cases (16.1%) were lost to follow-up.

### Factors affecting variable phenotypes and outcomes

One hundred and thirty-two clinical diagnosed patients were divided into two groups depending on whether they showed a developmental delay, epilepsy, hydrocephalus, and anemia. Factors including age at onset, age at treatment initiation, time from onset to treatment initiation, and biochemical metabolic markers at first visit were analyzed, except for two cases whose data was not available (Table 3). There was significant difference in the onset time, the age at treatment initiation and the time from onset to treatment initiation between developmental delay and non-developmental delay groups.

**Table 3:** Variable phenotypes and factors in 130 patients with *MMACHC* c. 609G > A homozygous mutation

Symptoms and signs		Cases	Age at onset	Age at treatment initiation	Time delays from onset to treatment initiation	Urinary methylmalonic acid pre-treatment	Blood tHcy pre-treatment	Blood methionine pre-treatment
			(months)	(months)	(months)	(mmol/mol creatinine)	(μmol/L)	(μmol/L)
		n	Median (P25, P75)	Median (P25, P75)	Median (P25, P75)	Median (P25, P75)	Median (P25, P75)	Median (P25, P75)
**Seizures**	N	65	3.0 (1.0, 15.0)	6.5 (2.0, 34.0)	1.5 (0.0, 18.3)	184.7 (43.5, 560.1)	99.8 (73.7, 129.6)	9.7 (7.0, 20.4)
	Y	65	2.0 (0.9, 5.0)	5.5 (3.0, 27.0)	2.3 (1.0, 12.0)	493.4 (259.9, 906.8)	96.0 (58.8, 151.7)	16.0 (10.4, 21.5)
Z			−1.407	−0.031	−0.893	−2.239	− 0.304	− 0.87
*P*			0.159	0. 975	0.372	**0.025**	0.761	0.406
**Developmental delay**	N	38	1.0 (0.7, 3.0)	3.0 (1.5, 5.5)	1.5 (0.9, 2.3)	164.6 (35.0, 664.9)	102.9 (65.3, 142.6)	16.0 (9.4, 20.4)
	Y	92	3.0 (1.0, 12)	12.0 (3.3, 36)	6.0 (0.5, 25.0)	443.6 (199.2, 722.1)	88.0 (59.1, 147.7)	10.4 (6.5, 22.6)
Z			−2.892	−3.746	− 2.017	−1.481	−0.313	− 0.707
*P*			**0.004**	**< 0.001**	**0.044**	0.14	0.754	0.503
**Hydrocephalus**	N	108	2.0 (1.0, 10.0)	8.0 (3.0, 34.0)	2.0 (0.5, 18.0)	282.1 (76.7, 768.1)	93.0 (63.0, 143.7)	14.0 (8.1, 20.4)
	Y	22	2.0 (1.0–4.0)	4.0 (2.8–0.5)	1.8 (0.9–6.8)	500.0 (257.3–560.1)	101.0 (59.1–161.9)	14.0 (5.7–21.5)
Z			−0.487	−0.92	− 0.531	− 0.608	−0.546	− 0.445
*P*			0.626	0.357	0.595	0.552	0.593	0.695
**Anemia**	N	93	3.0 (1.0, 7.8)	8.0 (3.0, 34)	3.0 (0.0, 24)	292.6 (92.3, 676.1)	87.2 (58.8, 127.1)	13.8 (7.4, 22.3)
	Y	37	2.0 (1.0, 5.0)	3.0 (2.0, 18.0)	1.5 (1.0, 9.0)	442.3 (97.0, 672.9)	137.3 (74.3, 164.9)	12.2 (6.7, 16.2)
Z			−0.622	−2.185	−0.777	−0.014	−2.172	− 0.64
*P*			0.534	0.029	0.437	0.989	**0.03**	0.56

Notes: *n* number, *N* No, *Y* Yes, *Z* Mann–Whitney U Z-score; *P* value was calculated by using a nonparametric unpaired Mann-Whitney U test. *P* < 0.05 was considered as statistically significant

In comparison with patients in the non-epileptic group, those in the epileptic group showed significantly higher levels of urinary methylmalonic acid. There were no significant differences in age at onset, age at treatment initiation, time from onset to treatment initiation, and biochemical metabolic markers (tHcy, methionine, free carnitine, acetylcarnitine, propionylcarnitine, propionylcarnitine/free carnitine ratio, and propionylcarnitine/acetylcarnitine ratio) between the groups (Table 3).

Forty-one patients with seizures were divided into two groups, seizure-free (*n* = 21) and uncontrolled (*n* = 20) groups, depending on whether they still had clinical seizures in the last 6 months (up to December 2019). As of December 2019, the age of the seizure-free group ranged from 1 year 5 months to 26 years 7 months (median 7 years 7 months). The age of the uncontrolled group ranged from 6 years to 16 years 3 months (median 10 years 8 months). The treatment recommendations for the two groups were the same. However, it is difficult to confirm whether the injection is missed because all the patients were given parenteral route of administration IM at home for long-term treatment. Some of the parents had poor compliance to frequent injection for their children. To reduce the plasma total homocysteine, the dose of cobalamin should be increased for the patients of uncontrolled group. General information pertaining to patients and recent condition were compared between the groups depending on whether clinical seizures had been controlled (Table 4).

**Table 4:** Data of 41 long-term follow-up patients caused by the *MMACHC* c.609G > A homozygous mutation with seizures

Group of the patients	Uncontrolled			Seizure-free			***Z***	***P***
	(***n*** = 20)			(***n*** = 21)				
	Median	P25	P75	Median	P25	P75		
Age at onset (months)	1	0.3	10.0	2	1.0	5.0	−0.756	0.472
Age at treatment initiation (months)	13	1.3	59.0	6.5	2.3	28.5	−0.047	0.982
Time from onset to treatment initiation (months)	8	1.3	52.0	5.5	0.7	23.3	−0.875	0.39
Latest urinary methylmalonic acid	20.5	6.1	75.0	16.5	12.4	47.9	−0.279	0.802
Latest blood metabolic markers								
Methionine (μmol/L)	17.1	14.9	23.5	15.7	12.8	27.1	−0.537	0.603
tHcy (μmol/L)	82.9	58.2	96.6	51.4	36.2	66.0	−2.413	**0.015**
Free carnitine (μmol/L)	56.8	40.0	77.7	56.6	27.6	69.2	−0.67	0.535
Acetylcarnitine (μmol/L)	26.1	14.8	33.2	23.5	9.9	28.3	−0.952	0.368
Propionylcarnitine (μmol/L)	5.4	3.7	7.7	3.7	0.8	6.5	−1.093	0.298
Propionylcarnitine/free carnitine ratio	0.09	0.08	0.12	0.08	0.06	0.18	−0.462	0.68
Propionylcarnitine/ acetylcarnitine ratio	0.27	0.18	0.31	0.2	0.19	0.25	−1.029	0.332

Notes: *n* number, *Z* Mann–Whitney U Z-score; *P* value was calculated by using a nonparametric unpaired Mann-Whitney U test. *P* < 0.05 was considered as statistically significant

During long-term follow-up, based on recent metabolic studies, we noted a significant difference in plasma total homocysteine (tHcy) levels among patients in the seizure-free and uncontrolled groups. Plasma tHcy in patients in the uncontrolled group were significantly higher than those in the seizure-free group. There were no significant differences in the age at onset, age at diagnosis, time from onset to diagnosis, latest urinary methylmalonic acid levels, blood free carnitine, acetylcarnitine, propionylcarnitine, propionylcarnitine/free carnitine ratio, and propionyl carnitine/acetylcarnitine ratio between the groups.

Skin lesions were present in 12 patients. Their blood methionine level was significantly low (5.6–10 μmol/L, normal control 12–50 μmol/L), and they presented with feeding difficulties, malnutrition, and failure to thrive. Five patients showed leucine, valine, and threonine deficiencies. Skin erosions occurred in three infants 3 to 5 days after feeding by medical formula for cobalamin non-responsive isolated methylmalonic aciduria. The condition of all patients improved soon after metabolic treatment with normal diet.

## Discussion

*CblC* deficiency caused by *MMACHC* mutation is the most common type of MMA combined with homocysteinemia. Although the genetic epidemiological data on *CblC* deficiency are limited, the carrier prevalence of the *MMACHC* mutation has been deduced to be 1/28–31 from the reported *cblC* incidences in the population in Shandong province, China [16, 17]. The c.609G > A pathogenic variant has been reported the hot spot mutation in Chinese patients with early onset *cblC* deficiency [18–20], however, the correlation of phenotype with the c.609G > A mutant allele has not been described.

In the present study, we investigated the clinical and biochemical characteristics of the patients with *cblC* deficiency caused by *MMACHC* c.609G > A homozygous mutation to discuss the optimal strategies of treatment and prevention. All of the 149 patients with the same genotype of c.609G > A homozygous alleles presented with significant differences in clinical phenotypes, ranging from asymptomatic condition to severe brain damage to death.

With early treatment, developmental milestones of the two prenatally diagnosed patients and the 10 patients detected by newborn screening were normal. No organ damage occurred. These cases benefited from early diagnosis, treatment, and prevention of severe complications. Fortunately, newborn screening for MMA is widely promoted around China in recent years, which has proven to be helpful to improve the outcome of patients with MMA [21, 22]. The five patients detected by newborn screening but treated after disease onset because of poor treatment compliance showed severe mental and motor retardation. Two of them had hydrocephalus, and one had seizures. A delay in treatment leads to irreversible brain damage, pointing out emphatically the pre-symptomatic treatment and education to improve parents’ understanding and medication compliance.

One hundred and thirty-two patients who were diagnosed after onset with the *MMACHC* c.609G > A homozygous mutation showed various symptoms. The most common symptoms were developmental delay, epilepsy, anemia, feeding difficulty and hydrocephalus. Brain damage is also commonly observed in patients with the *MMACHC* c.482G > A mutation [23]. Although most patients showed an improvement after metabolic treatment, there are varied neurological sequelae. The level of urinary methylmalonic acid was higher in patients with seizures pre-treatment, indicating an increase in toxic metabolites. Seizures have been reported to occur after injecting methylmalonic acid into the striatum of rats [24, 25]. Patients with no seizures show lower urinary methylmalonic acid levels, which is considered to be associated with severe brain damage in patients with higher levels of methylmalonic acid [26, 27]. It is more difficult to control seizures in patients with persistently high plasma tHcy levels. Excluding poor medication compliance, it is suggested that homocysteine may play a role in the pathogenesis. In vitro studies have suggested that homocysteine-induced phosphorylation disorder, overactivation of N-methyl-D-aspartate receptor [28], vascular oxidative stress, and inflammation [29] are related to neurological dysfunction [30]. Moreover, neurologic involvement in CblC patients was reported due to brain choline deficiency caused by the transmethylation defect [31]. There was also a small cohort study that found no significant correlation between plasma homocysteine or methylmalonic acid and the long-term prognosis of neurodevelopment [32]. Therefore, the relevant metabolic indicators still need to be further explored.

Methylcobalamin deficiency in patients with *cblC* deficiency is reportedly caused by elevated tHcy and reduced tetrahydrofolic acid levels. Impaired folic acid metabolism affects the synthesis of nucleotides [33]. In the 132 patients of this study, the main hematological abnormality was megaloblastic anemia, which is consistent with the results of a previous study [26]. At the first visit, blood tHcy levels in patients with anemia were higher than in those without anemia.

Eye diseases are common in patients with the *MMACHC* gene c.271dupA homozygous mutation and are characterized by early maculopathy. However, in this study, the incidence of ocular problems in patients with the c.609G > A homozygous mutation was 17.4%, which is much lower [34, 35]. It is considered that some patients didn’t undergo a detailed examination. An ophthalmological examination should be paid attention to in future diagnosis and treatment.

The *MMACHC* c.80A > G mutation is associated with pulmonary hypertension in patients with *cblC* deficiency [36]. It has been reported that c.271dupA, c.276G > T, and c.565C > A are more common in patients with renal thrombotic microangiopathy caused by MMA [37]. However, only two cases with proteinuria were observed in this study. After metabolic treatment, their proteinuria disappeared. Cardiovascular diseases have not been observed in patients with the c.609G > A homozygous mutation.

Skin lesion was found in 12 patients in this study. They are considered to be associated with essential amino acid deficiency due to malnutrition. In three cases, eczema and perineal erosion occurred after a low protein diet and taking the special medical formula for cobalamin non-responsive isolated methylmalonic aciduria. Nine patients had skin lesions and feeding difficulties at the onset of the disease. The condition of all patients improved soon after metabolic treatment with a normal diet.

In the present study, most (76.5%) patients with the *MMACHC* c.609G > A homozygous mutation showed early onset. The c.609G > A variant is nonsense and results in pre-mature termination codon, which is predicted to cause a truncated or abscent *MMACHC* protein. Similarly, two nonsense mutations, c.271dupA and c.331C > T, either homozygous or compound heterozygous, have been mainly reported in early onset patients; while c.394C > T and c.482G > A were reported to be related with late-onset patients [38]. This could be related to the low mRNA transcription level of the *MMACHC* gene and residual function of the enzyme [23, 39].

## Conclusion

We assessed a cohort of 149 patients with *cblC* deficiency caused by the *MMACHC* c.609G > A homozygous mutation to determine phenotypic differences in patients with the same genetic defect. Most patients showed early-onset, culminating in irreversible brain damage. Delayed treatment resulted in developmental delay. Newborn screening and early treatment are pivotal to prevent disabilities, however, some severe cases showed symptoms as early as a few minutes after birth. Newborn screening seemed to be late for very early onset patients. Moreover, given that a much higher carrier prevalence of the *MMACHC* c.609G > A mutation has been deduced in Chinese population, we propose that the c.609G > A mutant allele should thus be listed in the pre-pregnancy carrier screening panel in China.

## Data Availability

The datasets used and/or analyzed during the current study is available from the corresponding author on request.
